# Challenges of Measuring Self-Reported Exposure to Occupational Biomechanical Risk Factors Amongst People with Low Literacy Engaged in Manual Labour: Findings from a Cross-Cultural Adaptation and Psychometric Investigation in an African Population with Chronic Low Back Pain

**DOI:** 10.1007/s10926-024-10171-5

**Published:** 2024-02-20

**Authors:** Chinonso Nwamaka Igwesi-Chidobe, Isaac Olubunmi Sorinola, Benjamin Chukwuma Ozumba, Emma Louise Godfrey

**Affiliations:** 1 School of Allied Health Professions and Midwifery, Faculty of Health Studies, University of Bradford, Bradford, UK; 2 Global Population Health (GPH) Research Group, University of Nigeria, Nsukka, Nigeria; 3 School of Human Sciences, London Metropolitan University, London, UK; 4 Faculty of Medicine, College of Medicine, University of Nigeria (Enugu Campus), Enugu, Nigeria; 5 Department of Physiotherapy, School of Population Health Sciences, Faculty of Life Sciences and Medicine, King’s College London, London, UK; 6 Department of Psychology, Institute of Psychiatry, Psychology and Neuroscience, King’s College London, London, UK

**Keywords:** Occupational biomechanical risk factors, Low back pain, Cross-cultural adaptation, Psychometric evaluation, Rural and urban Nigeria, Low literacy

## Abstract

**Purpose:**

Occupational biomechanical factors are implicated in the aetiology and progression of low back pain (LBP). This study cross-culturally adapted and psychometrically investigated the Occupational Risk Factor Questionnaire (ORFQ) in a low literate Nigerian Igbo population with chronic LBP.

**Methods:**

Forward and back translation of the original ORFQ by clinical and non-clinical translators was followed by an expert committee review. The adapted ORFQ was pre-tested amongst rural Nigerian adults with chronic LBP using cognitive think-aloud interviewing. Internal consistency (Cronbach’s alpha) and test–retest reliability (unweighted and linear weighted k statistic for item-by-item agreement, and intra-class correlation coefficient—ICC) were investigated amongst 50 rural and urban Nigerian dwellers with chronic LBP. Spearman’s correlation and regression analyses were conducted with the Igbo-ORFQ, and measures of disability [World Health Organisation Disability Assessment Schedule (WHODAS 2.0), Roland Morris Disability Questionnaire (RMDQ), Back performance scale (BPS)], pain intensity [Eleven-point box scale (BS-11)] and social support [Multidimensional Scale of Perceived Social Support (MSPSS)], to test construct validity with 200 rural Nigerian dwellers with chronic LBP.

**Results:**

Cross-cultural adaptation highlighted difficulty conceptualising and concretising exposure to biomechanical risk factors. Item-by-item agreement, internal consistency (α = 0.84) and intraclass correlation coefficient (ICC = 0.83) were good. Some unexpected direction of associations between the biomechanical components of the Igbo-ORFQ, and disability, pain intensity, and social support prohibits establishment of construct validity.

**Conclusion:**

Prospective studies comparing the Igbo-ORFQ to other measures of exposure to occupational biomechanical risk factors are required to establish the construct validity of the Igbo-ORFQ.

**Supplementary Information:**

The online version contains supplementary material available at 10.1007/s10926-024-10171-5.

## Background

Biomechanical factors including increased and prolonged trunk flexion and twisting, and spinal loading are important in the aetiology and course of low back pain (LBP) [1–6]. However, there is conflicting evidence for dose-response relationships because of the different thresholds used in quantifying postural exposure and spinal loading in these studies. Repetitive or sustained spinal tissue loading is suggested to have a U-shaped relationship with spinal structures as very low or very high tissue load is believed to lead to spinal tissue injury, whilst moderate spinal loading is believed to be protective [1, 7]. However, the exact meaning of very low, moderate and very high tissue loading is ill-defined [3, 4, 6], and may vary in different individuals, making establishment of causal relationships difficult. Moreover, the ‘’healthy worker’’ effect, where healthy workers remain in their physically demanding jobs whereas less healthy workers are more likely to change or leave such jobs, has been shown to obscure the association between biomechanical factors and LBP [8]. There is insufficient evidence for posture, vibration, and driving, as prognostic factors for duration of sick leave because only a few studies have studied them [9].

A systematic review that investigated the psychosocial and physical factors associated with chronic LBP found that doing heavier work predicted longer duration of sick leave [9]. The influence of occupational biomechanical factors in chronic LBP has been suggested in other primary studies that accounted for psychosocial factors. For instance, a prospective study that examined individual, psychosocial, and workplace risk factors associated with the transition from acute to chronic occupational back pain found that severe leg pain, obesity, self-reported disability, psychological distress, the unavailability of light duties on return to work, and a job requirement of lifting for at least three quarters of the day were independent determinants of the transition of LBP to chronic LBP [10]. Another prospective study found that adverse employment outcomes such as leaving jobs and the inability to carry out normal duties depended more on the physical demands of a job such as lifting, bending, twisting, digging or shovelling [11]. In contrast, functional disability may be more strongly predicted by poor mental health and the tendency to somatise [11]. In another study, participants who changed to less heavy tasks were more likely to have a sustained remission of mild back pain symptoms, suggesting that heavy work may be associated with more LBP symptoms [12]. These results were independent of abnormal spinal structural findings, and heavy work was not associated with increased functional disability in the study. Sustained remission from baseline persistent back pain appeared to be linked to occupational factors including leaving a heavy labour occupation, neurophysiological variables such as chronic non-lumbar pain, and psychosocial factors such as psychological distress and fear avoidance beliefs in another prospective study [13].

Other studies have reported contradicting findings. In a French population predominantly involved in heavy jobs involving heavy lifting and repeated trunk flexion, combined biomechanical and psychosocial occupational exposures during working life appeared to have additive and interactive effects on functional health in retirement. Notably, only 38% of the workers had functional limitations suggesting that biomechanical factors were important in only a few of these individuals [14]. In another prospective cohort study, the physical demands of a job did not predict return to work status after accounting for pain intensity, workers’ compensation, female gender, personality, depression, and severity of injury [15]. However, this result could have been due to the exclusion of people who had left their previous jobs or were seeking new jobs from the analyses in the latter study. People engaged in heavy work, without psychological distress, who had low fear avoidance beliefs were more likely to be resilient to chronic LBP disability than people with lighter occupations [13]. However, people in lighter occupations had significantly greater baseline psychological distress, which may have confounded the results in this study [13]. Heavy work did not appear to be related to non-return to work in a different review [16]. However, workload assessment was imprecise in this review.

Cumulative exposure to occupational biomechanical factors has been suggested to be protective, although this could be due to the ‘’healthy worker effect’’, as unhealthy cohorts left their occupation over time [17]. This ‘’healthy worker effect’’ was also apparent in another prospective study that suggested that the longer a person stayed in a physically demanding job, the less likely it was for this person to develop LBP [12]. This could be because people who developed LBP may have already left the job [12], which may explain why biomechanical factors, such as heavy lifting and prolonged kneeling or squatting, were more likely to predict new onset LBP in newly employed workers [7].

Unfortunately, the evidence for the importance of these factors were derived from high income countries. Limited studies have explored the importance of occupational biomechanical alongside occupational psychosocial factors in first onset and chronic LBP in African countries including Nigeria. Qualitative studies in rural Nigeria suggest that occupational biomechanical factors may be important in the experience of chronic LBP in rural Nigeria [18, 19]. However, the exact influence of these factors on specific clinical and behavioural outcomes amongst low literate people with LBP in rural Nigeria is unclear, possibly due to lack of relevant outcome tools to measure them in these contexts.

The Occupational Risk Factor Questionnaire (ORFQ) [20] is one of the few outcome measures available for assessing occupational biomechanical and occupational psychosocial risk factors. This study aims to cross-culturally adapt and psychometrically evaluate the ORFQ (a self-reported measure of exposure to occupational biomechanical and occupational psychosocial risk factors) in a low literate Nigerian population with chronic LBP.

## Methods

### Study Designs

Translation, cultural adaptation, test-retest measurements, and cross-sectional psychometric evaluation of the Igbo version of the ORFQ amongst Igbo speaking populations with chronic LBP in rural and urban Nigeria.

### Outcome Measures

#### Occupational Risk Factor Questionnaire (ORFQ)

The ORFQ is a 25-item self-report questionnaire of exposure to occupational biomechanical and occupational psychosocial factors [20]. The first five (1–5) items measure work organisational factors such as work pressure and stress. The other (6–25) items assess exposure to occupational biomechanical factors such as bending, twisting, lifting, pulling, pushing, forceful movements and static postures like prolonged sitting, awkward postures, and whole-body vibrations. There is a first introductory question ‘please describe the main tasks of your job’ which is open, not numbered, and is not one of the 25 items in the questionnaire [20]. Items were originally designed to be analysed independently as categorical items. However, to enable statistical comparisons with multiple factors, we aimed to validate a total scoring of items 6–25, using two methods, to capture the total exposure to biomechanical risk factors. For the first method of total scoring, each item was scored based on the biomechanical thresholds reported to predict disabling LBP in an African population [21, 22] as was used in a previous study in this population [23]. A score of one was given to an item when the duration of exposure to the biomechanical factor was half the time or more, or when the frequency of exposure to the biomechanical factor was 11–30 times or more. An item was scored zero when exposure was less. This scoring method aligns with evidence suggesting that these high thresholds of biomechanical exposure would predict adverse LBP outcomes whilst lower exposures may be protective [9, 11, 24]. Possible scores ranged between 20 (maximum) and 0 (minimum) with greater scores reflecting higher exposure to biomechanical risk factors. The second method of total scoring was based on an incremental score of exposure to biomechanical risk factors. Although moderate exposures to biomechanical risk factors may be protective, different thresholds have been used to quantify low, moderate, and high exposure to biomechanical risk factors [1–7], which make the use of specific cut-off thresholds difficult. This incremental scoring involved assigning scores to each of the options for each questionnaire item. Items 6–22 had 6 options for each item ranging from ‘almost never’ (scored 1) to ‘almost all the time’ (scored 6), with middle options including ‘about 10% of the time’ (scored 2), ‘about 25% of the time’ (scored 3), ‘half the time’ (scored 4), and ‘about 75% of the time’ (scored 5). These options signified the percentage of time the activity was performed. The last 3 items (23–25) had 5 options for each of the items ranging from ‘almost never’ (scored 1) to ‘over 30 times an hour’ (scored 5), with middle options including ‘less than once an hour’ (scored 2), ‘1–10 times an hour’ (scored 3), and ‘11–30 times an hour’ (scored 4). These options denoted the number of times in an hour that the activity is performed. Possible scores ranged between 118 (maximum) and 20 (minimum) with greater scores reflecting higher exposure to occupational biomechanical risk factors. Each of the items in the psychosocial subsection of the ORFQ (1–5) and each of the biomechanical items (6–25) were also scored and statistically analysed independently, as was intended in the original measure.

#### World Health Organisation Disability Assessment Schedule (WHODAS 2.0)

The WHODAS 2.0 was selected because it is a comprehensive disability outcome tool that conceptualises disability within the biopsychosocial model, emphasising all six domains of disability: cognition, mobility, self-care, getting along with people, life activities and participation [25, 26]. Importantly, the measure includes work-related disability which has been linked to exposure to occupational biomechanical risk factors as previously highlighted. WHODAS 2.0 has good face and content validity, construct validity, internal consistency, test-retest reliability, and responsiveness. The Cronbach’s alpha ranges between 0.94 and 0.98; test-retest reliability ranges between 0.93 and 0.98; and sensitivity to change ranges between 0.46 and 1.38 [25]. The Igbo-WHODAS which has been cross-culturally adapted and validated for this population was used [27]. Igbo-WHODAS has excellent psychometric properties with good internal consistency (α = 0.75–0.97), intra-class correlation coefficients (ICC = 0.81–0.93), standard error of measurements (5.05–11.10), and minimal detectable change (13.99–30.77); with at least moderate correlations (rs ≥ 0.3) with performance-based disability, self-reported back pain specific disability and pain intensity, with no ceiling or floor effects [27]. The complex scoring method which is comparable across populations and conditions was used. This is an “item-response-theory” (IRT) based scoring that takes into consideration multiple levels of difficulty for each WHODAS 2.0 item. It involves summing recoded item scores in each domain, summing all six domain scores, and converting the summary score into a metric ranging from 0 (no disability) to 100 (full disability) [25]. The WHODAS 2.0 total score and the life activities subscale which includes work-related disability were used in this study.

#### Roland Morris Disability Questionnaire (RMDQ)

RMDQ was included because it is the most commonly used valid measure of LBP functional disability [28]. It is a twenty-four item back specific self-report measure with each item having possible scores of 0 or 1 [29]. A total maximum score of 24 signifies the highest possible disability level and 0 means no disability. It has good face and content validity, construct validity, internal consistency, test-retest reliability, and responsiveness [30]. It has Cronbach’s alpha ranging between 0.84 and 0.93; test-retest reliability ranging between 0.72 and 0.91; and a 2-3-point change from baseline is considered clinically important [30]. RMDQ conceptualises disability at the three levels of the ICF: body structures and function, activities and participation, and environmental factors. However, unlike the WHODAS 2.0, it places less emphasis on participation, and does not capture work-related outcomes [31]. The Igbo-RMDQ which has been validated in this population [32] was used. Igbo-RMDQ has Cronbach’s alpha of 0.91, test-retest reliability of 0.84, and moderately high correlations (*r* > 0.6) with performance-based disability and pain intensity, with no ceiling or floor effects [32].

### Back Performance Scale (BPS)

BPS is a back-specific performance-based measure of mobility-related limitation that is objectively scored by an assessor [33]. It involves participants performing five physical performance tests (sock test, pick-up test, roll-up test, finger-tip-to-floor test, and lift test) which involve mobility of the trunk [33]. Sock test involves participants simulating putting on a sock from sitting. Pick-up test involves picking up a piece of paper from the floor. Roll-up test entails rolling up slowly from supine lying to a long sitting position with the arms relaxed. In finger-tip-to-floor test, participant stands on the floor with feet 10 centimetres apart; bends forward with straight knees and tries to touch the floor with the fingertips. The distance between the floor and the fingertips is then measured in centimetres. For the lift test, a participant repeats lifting a 5-kilogram box from the floor to a 76 cm table and back to the floor for one minute. The number of lifts is recorded. Each of the five tests has scores ranging from 0 to 3 depending on the difficulty or ease with which they are performed. A total possible score of 15 signifies maximum disability whilst 0 means no disability [33]. The measure has good validity and reliability: internal consistency of 0.73, moderate correlations with self-reported disability (*r* = 0.454), and test-retest reliability of 0.91 [33–35].

#### Eleven-point Box Scale (BS-11)

The BS-11 is a single eleven-point numeric scale for pain intensity, with eleven numbers (0 to 10) surrounded by boxes. Zero represents ‘no pain’ and 10 represents ‘pain as bad as you can imagine’ or ‘worst pain imaginable’ [36–39]. It is easy to comprehend, administer, and the best pain intensity outcome tool for people with limited literacy [36]. The Igbo-BS-11 which has been validated in this population [40] was used.

### Multidimensional Scale of Perceived Social Support (MSPSS)

The MSPSS is a self-report measure of subjectively assessed social support [41]. It contains 12 items with three subscales assessing social support from family, friends or significant other. Each of these three subscales has four items, which is added to produce a total score ranging between 4 (minimum score) and 28 (maximum score). For a total scoring of these three subscales, the minimum score is 12, and the maximum score is 84. Higher values reflect a greater perceived social support. Each of the items has a 7-point Likert scale with values between 1 (strongly disagree) to 7 (strongly agree). The original MSPSS has an internal consistency of 0.88 and test–retest reliability of 0.85 [41, 42]. The Igbo version of the MSPSS [43] which has been validated in this population was used. The Igbo-MSPSS has an internal consistency of > 0.80 for the subscales and for the total scoring with test-retest reliability of 0.82 and correlations with social support outcome tools suggesting that it is a valid and reliable tool [43].

The battery of questionnaires above was interviewer-administered due to low literacy rates.

### Cross-cultural Adaptation of the ORFQ

#### Participants for Cross-cultural Adaptation

Clinical physiotherapists, non-clinical translators, and an expert committee were recruited for cross-cultural adaptation. Translators included four clinical physiotherapists and three non-clinical translators (Igbo linguistic expert, businesswoman and a civil servant). Physiotherapist translators had between five and twenty years of clinical experience at the time of this study and were all practising in Nigeria. Two English experts (health psychologist and academic physiotherapist) who were university academics in the United Kingdom, and two Igbo experts (clinical psychologist and clinical physiotherapist) who were academics in Nigeria made up the expert review committee.

Pre-testing (piloting) of the pre-final version of the Igbo ORFQ was done with a sub sample of 12 participants from a previous study in rural Nigeria [18]. These were participants who were living in rural communities in Enugu state. They were recruited due to convenience as they had indicated interest in participating in this study after the previous study [18]. Evidence-based guidelines suggest that a sample of 12 people is sufficient for cross-cultural adaptation [44]. They were informed about this study after which informed consent was obtained from those who indicated interest in participating prior to involvement in this study.

### Procedure for Cross-cultural Adaptation

**Fig. 1:**
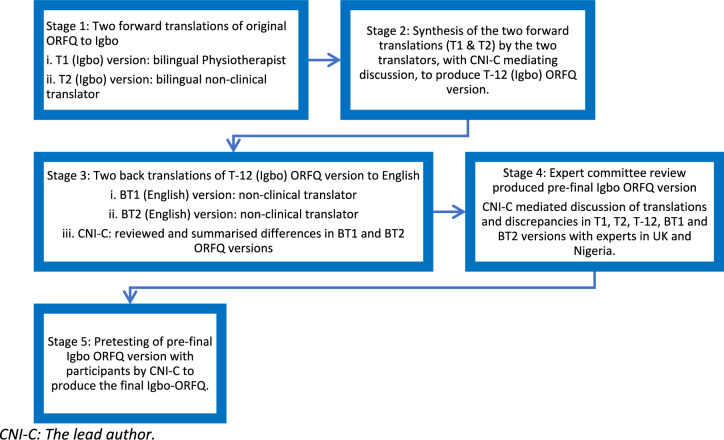
Cross-cultural adaptation procedure

Figure 1 below illustrates the evidence-based procedures [44] undertaken in the cross-cultural adaptation of the ORFQ. The ORFQ was forward translated from English to Igbo by one bilingual physiotherapist and one of the bilingual translators from a non-clinical background to produce the two Igbo ORFQ versions: T1 and T2 (Stage 1).

The T1 and T2 ORFQ versions were synthesised via discussion between the two forward translators, mediated by the lead author who is bilingual in English and Igbo. This produced one Igbo version: T-12. Translations were compared and discrepancies were documented (Stage 2).

The T-12 ORFQ version was back translated from Igbo to English by two of the non-clinical translators. This produced two back-translated English versions: BT1 and BT2. This process was a validation check which confirmed consistent translation, ensuring that the translated ORFQ version (T-12) was reflecting the construct in the original ORFQ (Stage 3).

All forward and back translated ORFQ versions (T1, T2, T-12, BT1, and BT2) were reviewed and critically appraised by the expert committee to produce the pre-final Igbo ORFQ version. The committee attempted to achieve cross-cultural equivalence via establishing semantic, idiomatic, experiential, and conceptual equivalence in Nigerian contexts [44]. For semantic equivalence, the committee explored Igbo and English words to determine whether they meant the same thing, if there were multiple meanings to an item, and if there were any grammatical difficulties in the translations. The committee ensured idiomatic equivalence by formulating alternative Igbo idioms and colloquialisms, where the English versions were difficult to translate or not applicable. Experiential equivalence was accomplished by the committee ensuring that questionnaire items were experienced similarly, for instance at the same level of intensity, in English and Igbo cultures. For conceptual equivalence, the committee determined that words in the items, instructions, and response options had similar conceptual meanings, that is, the ideas and principles are similarly interpreted in Igbo and English cultures. The expert committee also ensured that Igbo wordings were simple and could be easily understood by people with low literacy (Stage 4).

The pre-final Igbo ORFQ version was field tested amongst twelve participants living with chronic LBP in rural communities in Enugu state, who were part of a previous qualitative study [18]. The lead author interviewer-administered the measures using the ‘think-aloud’ cognitive interviewing procedure [45, 46]. This involved reading out each item of the questionnaire and asking participants to verbalise their thoughts as they were answering each question. The lead author asked participants if they found it difficult comprehending the pre-final Igbo ORFQ, what was understood by each item, and the meaning of the chosen response. The lead author encouraged participants to continue verbalising their thoughts as their responses was being recorded. This process was to ensure that equivalence was maintained in the target setting (Igbo culture in Nigeria) to confirm face and content validity. This final stage produced the final Igbo-ORFQ (Stage 5).

### Psychometric Evaluation of the Igbo-ORFQ

#### Participants and Sample Size

##### Participants

Participants were eligible if they were 18–69 years of age, had chronic LBP lasting for more than 12 weeks, had no underlying pathology associated with their pain such as malignancy, spinal fracture, infection, or cauda equina syndrome (LBP red flags), and were resident in the rural communities or urban communities in Enugu state, Nigeria. Participants were excluded if they were pregnant or had impaired capacity to give informed consent or to be interviewed which was identified via family reports or subjective assessment of speech coherence.

All eligible participants were stratified into males and females. Random selection by balloting (without replacement) was aimed at ensuring an equal representation of male and female participants.

### Sample Size for Reliability Investigation

A sample size of 50 is sufficient to detect a Kappa of 0.4 at 90% power for a proportion of positive ratings of 0.10–0.90 [47]. A convenience sample of 50 participants with chronic LBP aged 18–69 years were recruited from rural and urban communities in Enugu State, South-eastern Nigeria for test-retest reliability assessment. Informed consent was obtained prior to data collection.

### Sample Size for Construct Validity Evaluation

A sample size of 194 participants would give an 80% power to detect correlation coefficient of 0.2 at α level of 0.05. A sample size of 200 at a regression effect size (f2 = 0.366) at α of 0.05 with about 12 predictors indicates a 99.9% power to detect significant predictors [23]. Data for construct validity were collected from 200 participants who were representative of adults with chronic LBP in an Igbo Nigerian population who were living in rural communities in Enugu state, Nigeria, and were part of a previous study in which the process was described in detail [23].

Recruiting different samples of rural and urban Nigerian dwellers for aspects of the cross-cultural adaptation and psychometric evaluation was to guarantee a wide applicability of the Igbo-ORFQ across people with different levels of literacy in rural and urban Nigeria.

### Procedure

Ten community health workers (CHWs) were trained for interviewer-administration of the questionnaires due to low literacy rates in rural Nigeria. The research team decided to administer all the measures using interviewer-administration for all participants including the those that were literate to ensure consistency in data collection and avoid systematic differences in the results from different participants. Evidence suggests that interviewer-administration of self-report measures is valid when interviewers are adequately trained to minimise bias to patient responses [48, 49]. Moreover, interviewer-administration has been shown to reduce missing data [49], and may be the only way to administer self-report measures to people with limited literacy in low resource settings [50–52]. Training of the CHWs was done to prevent bias to participants’ responses and ensure that there were no missing items by ensuring that all questionnaire items were completed. Fidelity checks were completed during data collection to ensure that data collection was per protocol. Participants were screened first using screening questions that identified the LBP red flags. A body chart was subsequently used to confirm that pain was in the lower back. All the questionnaires were interviewer administered using flash cards to present the Likert scales as each item was read out to participants. The Igbo-ORFQ was completed at baseline, and then completed again 7–10 days to investigate test–retest reliability amongst the convenience sample of 50 rural and urban participants. The same CHW collected data on the two occasions. The Igbo versions of the questionnaires (ORFQ, WHODAS 2.0, RMDQ, BS-11, BPS, and MSPSS) were interviewer administered at one time-point in a cross-sectional design to investigate construct validity amongst the 200 rural dwellers. Different sample characteristics (rural and urban) was aimed at a wider applicability of the Igbo-ORFQ as well as the findings from this study.

### Statistical Analyses

Data were analysed with IBM SPSS version 22. The normality of data was determined using visual and statistical methods prior to data analyses.

#### Reliability

For test-retest reliability, item-by-item agreement was assessed with the k statistic for all the items of the Igbo-ORFQ. In this context, reliability was the extent to which the test-retest scores agreed, and not just the extent to which the test-retest scores were associated or correlated [47]. Median test-retest total scores with interquartile ranges (non-normally distributed data) were calculated in addition to identify the highest and lowest scores for the Igbo-ORFQ. Exposure to occupational biomechanical risk factors was expected to be stable over the short time interval between test-retest assessments. Unweighted and linearly weighted k statistic was calculated for each binary and ordinally scaled Igbo-ORFQ items respectively, as was done in the original measure [20]. Additional unweighted k statistic was calculated for items 6–25 which was adapted for total scoring. Zero indicated no agreement, 0.01–0.20 as none to slight, 0.21–0.40 as fair, 0.41– 0.60 as moderate, 0.61–0.80 as substantial, and 0.81–1.00 as almost perfect agreement [53]. In addition, intra-class correlation coefficient (ICC) was calculated for total scoring of the Igbo-ORFQ utilising a two-way random effects model (which has the underlying assumption that measurement errors could arise from either assessors or participants), using an absolute agreement definition between test-retest scores. Good, very good, and excellent ICCs were signified by 0.7, 0.8, and 0.9, respectively [54, 55]. Furthermore, internal consistency was assessed using Cronbach’s alpha to measure the extent to which the Igbo-ORFQ items measured the same or related construct, and was rated as low/weak (0-0.2), moderate (0.3–0.6), and strong (0.7-1.0) [56].

#### Validity

Construct validity evaluates the extent to which a measure assesses the construct it was intended to measure [56, 57]. Convergent validity, which assesses whether two tools that measure constructs that should be theoretically related, are indeed related [58, 59] and discriminant validity, which explains whether two constructs that should be theoretically unrelated, are in fact unrelated [55], both of which are domains of construct validity, were investigated using Spearman correlation coefficient (non-normally distributed data). This was graded as not relevant (< 0.10), low (0.10–0.30), moderate (0.30–0.50), and high (> 0.50) [43, 58, 60]. Furthermore, linear regression analyses were conducted with the biomechanical items of the ORFQ with each of the main outcomes of self-reported generic disability (Igbo-WHODAS), self-reported back pain specific disability (Igbo-RMDQ), back-specific performance-based mobility-related limitation (BPS), self-reported numeric pain intensity (BS-11), to clarify the amount of variance of these outcomes explained by one standard deviation change in each biomechanical factor. Factor analysis was not conducted in this study because exposure to biomechanical factor is an observable and directly measurable variable. Factor analysis is most relevant for measures of latent variables that are difficult to be assessed directly [61]. Moreover, the measure was originally developed for the items to be analysed independently as categorical items [20].

There was no existing Igbo measure of exposure to occupational biomechanical risk factors at the time of this study. Hence, construct validity was investigated using expected relationships between occupational biomechanical risk factors and LBP outcomes from the literature to propose a priori hypotheses. Evidence suggests that exposure to occupational biomechanical risk factors may be associated with aggravation or persistence of LBP symptoms, chronicity of LBP, sick leave, and leaving a job [9–13]. Therefore, the Igbo-ORFQ was expected to significantly demonstrate positive associations with WHODAS 2.0 which includes work-related and functional disability; RMDQ which captures functional disability and chronicity of LBP; BS-11 which captures pain symptoms; and BPS which captures performance-based mobility-related limitation. There is no evidence that exposure to occupational biomechanical risk factors is associated with social support amongst people with chronic LBP. Therefore, the Igbo-ORFQ is expected to have no significant correlation with the MSPSS measuring social support.

## Results

### Participants’ Characteristics

The tables below detail the sociodemographic characteristics of the participants that contributed to cross-cultural adaptation (Table 1), reliability testing (Table 2), and validity testing (Table 3) of the Igbo-ORFQ.

Education was measured as a categorical variable in the sample that pre-tested the measure (Table 1) who were part of a previous qualitative study in rural Nigeria [18]. However, education was measured as a continuous variable of years spent in education in the two samples for reliability (Table 2) and validity (Table 3) testing, and was measured using the socio-demographic section of the World Health Organisation Disability Assessment Schedule (WHODAS) [27]. Values less than six years means that primary education had not been completed. Six years means completion of primary school. 12 years means completion of secondary school. Values between 6 and 12 means completion of primary school and some years spent in secondary school which had not been completed. 16 years means completion of tertiary education. Values between 12 and 16 years means completion of secondary school and some years in tertiary education which had not been completed. Values above 16 years means professional tertiary education (such as Medicine, Law) or postgraduate level of education (such as Masters, PhD).

The characteristics of the three samples were broadly similar. All the participants were engaged in subsistence farming to different levels to support the feeding of their families. Majority were in addition either self-employed or in paid employment. The reliability sample which included urban and rural dwellers had higher educational levels and literacy rates than the cross-cultural adaptation and validity testing samples. Specifically, whilst half of the sample that pre-tested the measure (Table 1) had no education or primary level of education, and the mean years of education completed in the validity sample (Table 3) corresponded to completion of primary school; the reliability sample (Table 2) had mean years of education completed which corresponded to completion of secondary school.

**Table 1:** Demographic characteristics of participants that pre-tested the adapted ORFQ

*n* = 12	Frequency	%
Mean age = 45 years (SD 9.75)		
GENDER		
Male	7	58.33
Female	5	41.67
MAIN OCCUPATION		
Manual workers	7	58.33
Non-manual workers	5	41.67
RELIGION (CHRISTIAN DENOMINATION)		
Protestant Pentecostal	10	83.33
Catholic	2	16.67
MARITAL STATUS		
Married	11	91.67
Single	1	8.33
EDUCATIONAL LEVEL COMPLETED		
Secondary	4	33.33
Primary	3	25.00
None	3	25.00
Tertiary	2	16.67
LITERACY (ABILITY TO READ AND WRITE)		
Illiterate (inability to read and write)	4	33.33
English	6	50.00
English and Igbo	2	16.67

**Table 2:** Demographic characteristics of participants that contributed to reliability testing

*n* = 50	Frequency (%)	Mean (SD)
Gender		
Female	32 (64.0)	
Male	18 (36.0)	
Habitation		
Rural	20 (40.0)	
Urban	30 (60.0)	
Age (years)		45.2 (11.55)
Education (years)		13.3 (7.14)
Current marital status		
Currently married	37 (74.0)	
Never married	8 (16.0)	
Widowed	4 (8.0)	
Separated	1 (2.0)	
Work status		
Paid work	25 (50.0)	
Self-employed (own business including farming for commercial reasons)	19 (38.0)	
Keeping house/homemaker	2 (4.0)	
Student	2 (4.0)	
Non-paid work (volunteer or charity)	1 (2.0)	
Unemployed (health reasons)	1 (2.0)	

**Table 3:** Demographic characteristics of participants that contributed to validity testing

*n* = 200	n (%)	Mean (SD)
Sex		
Female	112 (56.0)	
Male	88 (44.0)	
Age (years)		48.6 (12.0)
Education (years)		7.0 (6.4)
Current marital status		
Currently married	143 (71.5)	
Widowed	31 (15.5)	
Never married	22 (11.0)	
Cohabiting	2 (1.0)	
Separated	2 (1.0)	
Work status		
Self-employed (own business or farming)	125 (62.5)	
Paid work	31 (15.5)	
Non-paid work (volunteer or charity)	16 (8.0)	
Keeping house/homemaker	13 (6.5)	
Student	7 (3.5)	
Unemployed (health reasons)	4 (2.0)	
Unemployed (other reasons)	3 (1.5)	
Retired	1 (0.5)	

### Findings from the cross-cultural Adaptation of the ORFQ

There were no major disagreements between forward and back translators. There is no indigenous Igbo word for ‘percentage’ in the response options of the ORFQ. This was spelt in Igbo as ‘pacenti’, in line with Igbo grammatical expression [62, 63]. During the pre-testing stage, it was found that people with limited literacy found it hard to conceptualise the intensity of exposure to biomechanical risk factors in terms of percentage. Therefore, the suggestion of the expert review committee led to the inclusion of an additional statement explaining these percentages of exposure in terms of concrete numbers such as the number of work days per week to enhance understanding. Similarly, for item 8, there is no indigenous Igbo word for ‘…degrees’ which was spelt as ‘digrii’, in line with Igbo grammatical expression [64]. ‘Kilograms’ was added to statements with ‘pounds’ as this is a more familiar measurement unit in Nigeria. Even so, illiterate rural dwellers struggled to think in abstract terms using the kilograms. Therefore, the weights of objects commonly used in this environment corresponding to the weights named in the ORFQ items such as gallons of water were added to align with the expert review committee recommendations. Furthermore, it was decided during the expert committee review that in addition to reading out the Igbo-ORFQ items to participants, each movement/activity would be demonstrated by the interviewer to ensure comprehension by all. Participants found this useful during the verbal pre-testing. See Appendix 1 for the Igbo-ORFQ.

### Psychometric Properties of the Igbo-ORFQ

#### Reliability of the Igbo-ORFQ

Table 4 below shows that the unweighted and linear weighted Kappa were fair to almost perfect for all items except items 19 (K = 0.23, *p* > 0.05) and 24 (K = 0.25, *p* > 0.05). Internal consistency (α = 0.84) and intraclass correlation coefficient (ICC = 0.83) were good for Igbo-ORFQ. Median test-retest scores for adapted total scoring of Igbo-ORFQ were the same.

**Table 4:** Reliability of Igbo-ORFQ

**Items**	**Done in the original measure**	
	**Unweighted K **	**SE**
1	0.65**	0.13
2	0.34*	0.14
3	0.34*	0.18
4	0.41*	0.14
5	0.54**	0.13

**Adapted for this study**	**Done in the original measure**			
	**Linear weighted K **			**SE**
6	0.52**	0.14	0.52	0.09
7	0.65**	0.14	0.56	0.10
8	0.56**	0.13	0.51	0.09
1b	0.55**	0.15	0.52	0.10
2b	0.81**	0.11	0.49	0.10
3b	0.50**	0.15	0.59	0.09
4b	0.58**	0.14	0.57	0.09
5b	0.43*	0.16	0.50	0.10
6b	0.42*	0.15	0.32	0.09
7b	0.41*	0.17	0.49	0.10
8b	0.57**	0.11	0.47	0.08
9	0.50**	0.15	0.34	0.11
10	0.46*	0.23	0.46	0.11
11	0.23∞	0.23	0.37	0.13
12	0.64**	0.19	0.42	0.09
13	0.38*	0.27	0.35	0.11
14	0.38*	0.27	0.36	0.14
15	0.60**	0.15	0.45	0.10
16	0.25∞	0.19	0.36	0.09
17	0.56**	0.20	0.54	0.10

	Total scoring^ɒ^	Total scoring⊕
Cronbach’s alpha	0.84	0.87
ICC	0.83	0.86
Median (IQR) test	2.00 (1, 5)	47.00 (40, 58.25)
Median (IQR) retest	2.00 (1, 5)	47.00 (40, 58.25)
Minimum scores	0	22
Maximum scores	19	118

** Significant at p<0.0005; *Significant at p<0.05; K= kappa statistic; SE= standard error; ɒ = adapted total scoring with the specific threshold for biomechanical exposure; ⊕ = adapted total scoring using incremental exposure to biomechanical risk factors; ∞= not significant

#### Construct Validity of the Igbo-ORFQ

Tables 5 and 6 highlight the construct validity of the Igbo-ORFQ. Table 5 below shows that the total score of the Igbo-ORFQ using the threshold scoring method had significant positive correlations with the Igbo-MSPSS total score and all its subscales. The Igbo-ORFQ total score using the incremental scoring system had a significant positive correlation with only the significant other subscale of the Igbo-MSPSS. The total score of the Igbo-ORFQ using the threshold scoring method had significant negative correlations with the Igbo-WHODAS total score and all its subscales, Igbo RMDQ (barely significant), and pain intensity. The Igbo-ORFQ total score using the incremental scoring system had a significant negative correlation with the Igbo-WHODAS total score and all its subscales, Igbo RMDQ, BPS, and pain intensity. Table 6 shows that most biomechanical risk factor items were either significantly and negatively associated with disability and pain intensity or had no significant association with disability and pain intensity. The biomechanical risk factor items with significant negative associations with self-reported generic disability (Igbo-WHODAS) were ORFQ6 (amount of time spent bending the trunk forward slightly, hands above knee level), ORFQ8 (amount of time spent twisting the trunk [over 45 degrees] and bending sideways), ORFQ2b (amount of time spent carrying loads with one hand), ORFQ8b (amount of time spent sitting), ORFQ12 (amount of time spent driving or riding motor vehicles), and ORFQ14 (amount of time spent working on elevated surfaces). The biomechanical risk factor items with significant negative associations with self-reported back pain specific disability (Igbo-RMDQ) were ORFQ4b (amount of time spent pushing/pulling loads), ORFQ7b (amount of time spent carrying loads over 10 pounds more than 40 feet), and ORFQ12 (amount of time spent driving or riding motor vehicles). The biomechanical risk factor item with significant negative association with back-specific performance-based mobility-related limitation (BPS) was ORFQ12 (amount of time spent driving or riding motor vehicles). The biomechanical risk factor item with significant negative association with self-reported numeric pain intensity (BS-11) was ORFQ17 (how often do you have to lift an object that weighs more than 30 pounds).

Only two biomechanical risk factor items had significant positive associations with self-reported generic disability (Igbo-WHODAS). These were ORFQ3b (amount of time spent handling objects difficult to grip-unstable, no handles) and ORFQ5b (amount of time spent carrying objects of 10–30 pounds). Only one biomechanical risk factor item had a significant positive association with self-reported back pain specific disability (Igbo-RMDQ). This was ORFQ16 (how often do you have to lift an object that weighs between 10 and 30 pounds).

**Table 5:** Spearman’s correlation between the Igbo-ORFQ (total scores), and the subscales and total scores of self-reported generic disability (Igbo-WHODAS), self-reported back pain specific disability (Igbo-RMDQ), back-specific performance-based mobility-related limitation (BPS), self-reported numeric pain intensity (BS-11), and self-reported social support (Igbo-MSPSS)

	Igbo-ORFQ^ɒ^ (p-value)	Igbo-ORFQ^⊕^ (p-value)
Igbo-WHODAS (Total)	− 0.383 (< 0.001)	− 0.367 (< 0.001)
Igbo-WHODAS (Cognition)	− 0.338 (< 0.001)	− 0.270 (< 0.001)
Igbo-WHODAS (Mobility)	− 0.320 (< 0.001)	− 0.353 (< 0.001)
Igbo-WHODAS (Self-care)	− 0.289 (< 0.001)	− 0.250 (< 0.001)
Igbo-WHODAS (Getting along with people)	− 0.357 (< 0.001)	− 0.330 (< 0.001)
Igbo-WHODAS (Life activities)	− 0.300 (< 0.001)	− 0.276 (< 0.001)
Igbo-WHODAS (Participation)	− 0.302 (< 0.001)	− 0.320 (< 0.001)
Igbo-RMDQ	− 0.145 (0.041)	− 0.353 (< 0.001)
BPS	− 0.058 (0.412)	− 0.207 (0.003)
BS-11	− 0.191 (0.007)	− 0.299 (< 0.001)
Igbo-MSPSS (Total)	0.226 (0.001)	0.125 (0.077)
Igbo-MSPSS (Family)	0.214 (0.002)	0.062 (0.383)
Igbo-MSPSS (Friends)	0.151 (0.032)	0.128 (0.071)
Igbo-MSPSS (Significant other)	0.265 (< 0.001)	0.157 (0.026)

ɒ = adapted total scoring with the specific threshold for biomechanical exposure; ^**⊕**^= adapted total scoring using incremental exposure to biomechanical risk factors; p significant at < 0.05

**Table 6:** Linear regression analyses between the biomechanical items of the Igbo-ORFQ and each of self-reported generic disability (Igbo-WHODAS), self-reported back pain specific disability (Igbo-RMDQ), back-specific performance-based mobility-related limitation (BPS), and self-reported numeric pain intensity (BS-11)

Igbo-ORFQ biomechanical risk factor items	Igbo-WHODAS β (p value)	Igbo-RMDQ β (p value)	BPS β (p value)	BS-11 β (p value)
ORFQ6	− 0.190 (0.011)	− 0.062 (0.444)	− 0.013 (0.873)	− 0.022 (0.792)
ORFQ7	0.050 (0.578)	0.033 (0.735)	0.153 (0.135)	0.047 (0.645)
ORFQ8	− 0.237 (0.007)	0.026 (0.782)	− 0.085 (0.387)	− 0.095 (0.339)
ORFQ1b	0.019 (0.835)	0.060 (0.541)	0.013 (0.894)	0.163 (0.111)
ORFQ2b	− 0.163 (0.043)	− 0.150 (0.091)	− 0.101 (0.265)	− 0.141 (0.126)
ORFQ3b	0.309 (*p* < 0.0001)	0.035 (0.693)	− 0.120 (0.185)	0.095 (0.295)
ORFQ4b	− 0.041 (0.636)	− 0.204 (0.033)	− 0.084 (0.394)	− 0.059 (0.551)
ORFQ5b	0.248 (0.018)	0.181 (0.117)	0.220 (0.064)	0.137 (0.253)
ORFQ6b	− 0.116 (0.280)	0.084 (0.477)	0.110 (0.369)	0.068 (0.579)
ORFQ7b	− 0.121 (0.174)	− 0.257 (0.009)	− 0.129 (0.201)	− 0.124 (0.224)
ORFQ8b	− 0.204 (0.003)	− 0.039 (0.591)	0.074 (0.328)	− 0.125 (0.103)
ORFQ9b	− 0.102 (0.212)	− 0.063 (0.481)	− 0.066 (0.475)	− 0.072 (0.442)
ORFQ10	0.074 (0.436)	− 0.002 (0.985)	− 0.066 (0.535)	0.059 (0.582)
ORFQ11	0.104 (0.144)	0.108 (0.170)	0.121 (0.133)	− 0.004 (0.965)
ORFQ12	− 0.163 (0.022)	− 0.170 (0.030)	− 0.305 (*p* < 0.0001)	− 0.127 (0.118)
ORFQ13	− 0.012 (0.864)	0.054 (0.494)	0.027 (0.741)	− 0.060 (0.467)
ORFQ14	− 0.199 (0.039)	− 0.181 (0.088)	0.035 (0.750)	− 0.170 (0.123)
ORFQ15	0.085 (0.324)	− 0.014 (0.886)	0.031 (0.752)	0.092 (0.353)
ORFQ16	0.074 (0.378)	0.202 (0.030)	0.069 (0.466)	0.106 (0.272)
ORFQ17	− 0.142 (0.136)	− 0.174 (0.095)	− 0.143 (0.182)	− 0.277 (0.011)

β = standardised beta

## Discussion

No cross-cultural adaptation of the ORFQ existed at the time that this study was conducted. Studies in low- and middle-income countries have either used the original measure [22, 65], developed new measures based on the content of the ORFQ [66], or used other measures of exposure to occupational biomechanical risk factors [67–69]. The difficulty in its cultural adaptation for rural Nigeria was related to the use of technical words such as ‘percentage’, ‘degrees’ and ‘pounds’ which did not have Igbo equivalents. Face and content validity could not be established using the items as they were in the original ORFQ because people with low literacy found it challenging to conceptualise the items. Content validity is an estimate of the validity of an outcome tool based on a detailed examination of the contents of the test items by subject experts and people with lived relevant experience; whereas face validity is an estimate by an experienced panel reviewing the content of an assessment or tool to see if it seems appropriate and relevant to the concept it purports to measure [70]. Therefore, the face and content validity of the Igbo-ORFQ was established by using concrete numbers such as the number of workdays per week in place of percentage of exposure, and the weights of objects commonly used in this environment in place of pounds and kilograms; whilst retaining the original items for use amongst literate people in this population in future studies. Face and content validity of the Igbo-ORFQ was further enhanced by the demonstration of the activities in the items during the interviewer-administration of the questionnaire by the CHWs. These processes enhanced understanding and acceptability whilst retaining conceptual equivalence. These limitations of the original ORFQ, which was addressed during the cross-cultural adaptation of the Igbo-ORFQ, does not appear to be present in other measures in which exposure to occupational biomechanical risk factors were expressed in concrete terms [67–69]. For instance, the Job requirements and physical demands (JRPD) questionnaire used specific number of hours of exposure rather than the percentage of exposure that was used in the original ORFQ [67, 68]. Another self-completed questionnaire measuring physical demands of work used specific duration of exposure in minutes and weights in kilograms [69].

The total scoring of the Igbo-ORFQ demonstrated good reliability with an internal consistency of 0.84 and intraclass correlation coefficient of 0.83. Unweighted and linear weighted kappa showed agreement for most Igbo-ORFQ items. In contrast to the original ORFQ, items 19 (operating powered hand tools) and 24 (lifting 10–30 pounds’ objects), did not show agreement, suggesting that these activities were not consistently performed in this population. Similar median test and retest scores for the total scoring of Igbo-ORFQ suggest the stability of the construct – exposure to occupational biomechanical risk factors and the Igbo-ORFQ in this population [71, 72].

The findings from the construct validity investigation were unexpected and contradicted the a priori proposed hypotheses. The results suggested that there were no major differences between the threshold and incremental total scoring methods, particularly in the direction of relationships. The strength of associations appeared to be stronger with the threshold total scoring for some outcomes and stronger with the incremental total scoring method for some other outcomes. Notably, the results showed significant negative associations between the total score of the Igbo-ORFQ, and disability and pain intensity. This suggests that the greater the exposure to occupational biomechanical risk factors, the less the work-related disability, functional disability, performance-based mobility related disability, and pain intensity. The cross-sectional design of the validity testing suggests that this relationship could either be causal or consequential. This directly contradicts the accounts of people with chronic LBP from a previous qualitative study in rural Nigeria [18] such as “*Now if I don’t go to work and perform my manual heavy duties for one, two, three weeks, I don’t have back pain. I will be normal. Like I’m sitting down now, I’m feeling the pains because I’m just from work but if I don’t go to work, I don’t have back pain*”. These results also contradict the findings from the validation of other questionnaires measuring exposure to occupational biomechanical risk factors [67–69]. For instance, the JRPD had significant but small positive correlations with pain intensity, perceived exertion, presence of symptoms, and functional limitations [67, 68].

Population characteristics including socio-economic and socio-cultural factors might explain some of these unexpected findings. Due to the cross-sectional design of the validity testing in the current study, negative associations between exposure to occupational biomechanical risk factors, and disability and pain intensity could also mean that the less the pain and disability, the more the people were able to engage in their manual jobs which was the predominant occupation. This suggests that increased exposure to occupational biomechanical risk factors was reflecting people that were more involved in their occupational work, which may have been a consequence of having less pain and disability. Therefore, it is possible that individuals in this population stayed off work when they were in pain and increased their level of manual work after recovering and returning to work. This might have been implied in previous studies in this population [18, 23].

Another explanation for these unexpected results could be related to the healthy worker effect. The rural Nigerian population that validated the Igbo-ORFQ were either farmers or combined farming with other informal manual jobs. This might have implied that higher exposure to occupational biomechanical factors were reflecting those in work, and lower exposures were reflecting those who were no longer working, perhaps temporarily, as there were limited non-manual jobs in this population. This aligns with prospective research evidence in high income countries suggesting that the longer people stayed in physically demanding jobs, the less likely it was for them to develop LBP because people who developed LBP had left the job earlier [12]. This aligns with the finding that exposure to biomechanical risk factors is more likely to predict new onset LBP in newly employed workers [7]. In contrast, the participants in the current study had chronic LBP. The construct of functional disability captured by the three disability outcome measures (Igbo-WHODAS, Igbo-RMDQ and BPS) reflects mobility related activities that were also commonly performed in the informal manual jobs that the participants were engaged in. This aligns with qualitative research evidence from this population [18, 19].

In view of these findings, other outcomes apart from disability and pain intensity, such as exposure to occupational biomechanical risk factors using other outcome tools and current episode(s) of LBP, using prospective rather than cross-sectional study designs, may help to expose the true relationships between occupational biomechanical factors and LBP outcomes in this population. Moreover, suggestions from our recently completed, yet unpublished studies, suggest that exposure to biomechanical risk factors may be better measured in this population by using simplified questionnaires that combine objective and subjective procedures. This included objective assessment of weight carried, and the frequency and duration that the weight was carried.

Further exploration of individual biomechanical items exposed interesting relationships with disability and pain intensity. Majority of the biomechanical items either had significant negative associations or no associations with disability and pain intensity, reflecting the findings from the total scoring of the Igbo-ORFQ. The biomechanical factors with negative associations with disability and pain intensity included postural risk factors such as bending, and stationary activities including sitting and driving. It is possible that these factors are either protective or unimportant for LBP outcomes in this population.

Interestingly, three biomechanical risk factor items had significant positive associations with disability and pain intensity. These items were the amount of time spent handling objects that are difficult to grip, amount of time spent carrying heavy weights (10–30 pounds), and the frequency of carrying heavy weights (10–30 pounds). These results suggest that spinal loading may be more important than the other occupational biomechanical risk factors captured in the ORFQ in explaining pain-related disability amongst people with chronic LBP.

The finding that the higher the exposure to occupational biomechanical risk factors from the total scoring of the Igbo-ORFQ, the more the social support could be reflecting the informal self-employed nature of most of the jobs undertaken by the people in this population. These manual jobs which included subsistence farming and family businesses often involved family members undertaking some manual activities entailed in the job as suggested by previous qualitative research evidence in this population [18, 19]. Therefore, jobs requiring more manual activities were more likely to involve more support from family members and friends to help with these activities. This might explain the positive association between exposure to occupational biomechanical risk factors and social support. There are reports that disability from LBP is greatest in the working population in low- and middle-income countries because the predominant informal employment preclude the feasibility of occupational modification [73–75]. Social support has also been found to be a consequence of mobility limitation, and could represent coping assistance given to people living with chronic LBP in this population [23].

Another possible but unlikely reason for these unexpected associations could be the difficulty in administering the ORFQ to people with low literacy, as well as reference to specific occupational activities which are not common in this population such as ‘operating powered hand tools’, ‘driving or riding motor vehicles’, ‘working on elevated surfaces, e.g., scaffold’. However, attempts were made to ameliorate this during the cross-cultural adaptation processes by concretising the items in the questionnaire as previously described.

However, the Igbo-ORFQ may not be clinically useful because it was originally developed as an epidemiological outcome measure. As the test-retest median scores from this study suggest, the Igbo-ORFQ is stable and may not be helpful in determining the impact of interventions by detecting changes in exposure arising from clinical, rehabilitation or public health interventions. This is more so for changes that arise from individual personal changes rather than job modifications which may not be possible in this lower middle-income country with minimal enforcement of occupational health regulations. Calls have been made to develop and deliver pragmatic interventions that target the factors associated with LBP outcomes in low- and middle-income countries [75]. Therefore, the findings from this study may inform the development of simple outcome tools that are sensitive to change to measure exposure to biomechanical risk factors within occupational as well as non-occupational settings.

The Igbo-ORFQ (Appendix 1), which is the complete questionnaire, can enable future studies to investigate the relative importance of occupational biomechanical factors and occupational psychosocial factors in explaining LBP outcomes.

This study is limited by the lack of bilingual investigation of item-by-item agreement between the original ORFQ and the Igbo-ORFQ because of the limited literacy of the participants. Furthermore, interviewer-administration of the measure by several CHWs could have introduced random or systematic bias in the measurements. The limited literacy of some participants could have affected the understanding of the items of the Igbo-ORFQ which could have biased the results. However, efforts were made to ameliorate this by concretising the items as previously described. Another limitation of this study is the lack of measurement of exposure to non-occupational biomechanical risk factors involving household or community work which may have biased the participants’ self-perceived level of exposure to occupational biomechanical risk factors. Additionally, the lack of measurement of participants’ work experiences including years of work, previous sick leave, and previous injuries make it difficult to ascertain the presence or absence of the healthy worker effect in this study. The lack of detailed assessment of job characteristics such as full-time or part-time work, shift patterns if present, working hours per day or week, which may affect self-perceived physical job demands and perceived exposure to occupational biomechanical and psychosocial risk factors are other limitations of this study. Finally, the inability to validate the Igbo-ORFQ with another measure of exposure to occupational biomechanical risk factors limit the establishment of construct validity. Future studies involving the Igbo-ORFQ could be designed addressing these limitations.

## Conclusion

The Igbo-ORFQ is an outcome measure of exposure to occupational biomechanical and occupational psychosocial risk factors. This tool requires further testing using prospective study designs. Furthermore, utilising a broader spectrum of outcome tools including those that measure biomechanical risk factors such as the JRPD to establish the construct validity of the Igbo-ORFQ in this population is required. However, the Igbo-ORFQ may not be sensitive to change and was not designed to identify the impact of clinical and public health interventions.

## Supplementary Information

Below is the link to the electronic supplementary material.

Supplementary material 1 (DOCX 23.9 kb)

## Data Availability

The datasets generated during the current study are available from the corresponding author on reasonable request.

## References

[1] Ribeiro DC, Aldabe D, Abbott JH, et al. Dose–response relationship between work-related cumulative postural exposure and low back pain: a systematic review. Ann Occup Hyg. 2012;56:684–96. 22356808 10.1093/annhyg/mes003

[2] Wai EK, Roffey DM, Bishop P, et al. Causal assessment of occupational bending or twisting and low back pain: results of a systematic review. Spine J. 2010;10:76–88. 19631589 10.1016/j.spinee.2009.06.005

[3] Bakker EWP, Verhagen AP, van Trijffel E, et al. Spinal mechanical load as a risk factor for low back pain: a systematic review of prospective cohort studies. Spine (Phila Pa 1976). 2009;34:E281–93. 19365237 10.1097/BRS.0b013e318195b257

[4] Waters T, Yeung S, Genaidy A, et al. Cumulative spinal loading exposure methods for manual material handling tasks. Part 1: is cumulative spinal loading associated with lower back disorders? Theor Issues Ergon Sci. 2006;7:113–30.

[5] Kwon BK, Roffey DM, Bishop PB, et al. Systematic review: occupational physical activity and low back pain. Occup Med (Chic Ill). 2011;61:541–8. 10.1093/occmed/kqr092 21727180

[6] Da Costa BR, Vieira ER. Risk factors for work-related musculoskeletal disorders: a systematic review of recent longitudinal studies. Am J Ind Med. 2010;53:285–323. 19753591 10.1002/ajim.20750

[7] Harkness EF, MacFarlane GJ, Nahit ES, et al. Risk factors for new-onset low back pain amongst cohorts of newly employed workers. Rheumatology. 2003;42:959–68. 12730508 10.1093/rheumatology/keg265

[8] Hartvigsen J, Bakketeig LS, Leboeuf-Yde C, et al. The association between physical workload and low back pain clouded by the healthy worker effect: population-based cross-sectional and 5-year prospective questionnaire study. Spine (Phila Pa 1976). 2001;26:1788–92. 11493851 10.1097/00007632-200108150-00011

[9] Steenstra IA, Verbeek JH, Heymans MW, et al. Prognostic factors for duration of sick leave in patients sick listed with acute low back pain: a systematic review of the literature. Occup Environ Med. 2005;62:851–60. 16299094 10.1136/oem.2004.015842 PMC1740930

[10] Fransen M, Woodward M, Norton R, et al. Risk factors associated with the transition from acute to chronic occupational back pain. Spine (Phila Pa 1976). 2002;27:92–8. 11805644 10.1097/00007632-200201010-00022

[11] McNee P, Shambrook J, Harris EC, et al. Predictors of long-term pain and disability in patients with low back pain investigated by magnetic resonance imaging: a longitudinal study. BMC Musculoskelet Disord. 2011;12:1–12. 21999666 10.1186/1471-2474-12-234 PMC3219563

[12] Adams MA, Mannion AF, Dolan P. Personal risk factors for first-time low back pain. Spine (Phila Pa 1976). 1999;24:2497. 10626313 10.1097/00007632-199912010-00012

[13] Carragee EJ, Alamin TF, Miller JL, et al. Discographic, MRI and psychosocial determinants of low back pain disability and remission: a prospective study in subjects with benign persistent back pain. Spine J. 2005;5:24–35. 15653082 10.1016/j.spinee.2004.05.250

[14] Sabbath EL, Glymour MM, Descatha A, et al. Biomechanical and psychosocial occupational exposures: joint predictors of post-retirement functional health in the French GAZEL cohort. Adv Life Course Res. 2013;18:235–43. 24796708 10.1016/j.alcr.2013.07.002

[15] Gatchel RJ, Polatin PB, Mayer TG. The dominant role of psychosocial risk factors in the development of chronic low back pain disability. Spine (Phila Pa 1976). 1995;20:2702–9. 8747248 10.1097/00007632-199512150-00011

[16] Truchon M, Fillion L. Biopsychosocial determinants of chronic disability and low-back pain: a review. J Occup Rehabil. 2000;10:117–42.

[17] Macfarlane GJ, Thomas E, Papageorgiou AC, et al. Employment and physical work activities as predictors of future low back pain. Spine (Phila Pa 1976). 1997;22:1143–9. 9160474 10.1097/00007632-199705150-00015

[18] Igwesi-Chidobe CN, Kitchen S, Sorinola IO et al. A life of living death: the experiences of people living with chronic low back pain in rural Nigeria. *Disabil Rehabil*; 39. Epub ahead of print 2017. 10.3109/09638288.2016.1161844. 10.3109/09638288.2016.1161844 27111492

[19] Igwesi-Chidobe CN, Sorinola IO, Kitchen S et al. Unconventional practitioners’ causal beliefs and treatment strategies for chronic low back Pain in Rural Nigeria. Heal Serv Insights; 11. Epub ahead of print 2018. 10.1177/1178632918808783. 10.1177/1178632918808783 PMC6207981 30397385

[20] Halpern M, Hiebert R, Nordin M, et al. The test–retest reliability of a new occupational risk factor questionnaire for outcome studies of low back pain. Appl Ergon. 2001;32:39–46. 11209830 10.1016/s0003-6870(00)00045-4

[21] van Vuuren B, Van Heerden HJ, Becker PJ, et al. Lower back problems and work-related risks in a South African manganese factory. J Occup réhabilitation. 2007;17:199–211. 10.1007/s10926-007-9073-4 17333380

[22] Van Vuuren BJ, Becker PJ, Van Heerden HJ, et al. Lower back problems and occupational risk factors in a South African steel industry. Am J Ind Med. 2005;47:451–7. 15828071 10.1002/ajim.20164

[23] Igwesi-Chidobe CN, Coker B, Onwasigwe CN et al. Biopsychosocial factors associated with chronic low back pain disability in rural Nigeria: a population-based crosssectional study. BMJ Glob Health. 2017;2(3):e000284. 10.1136/bmjgh-2017-000284. 29225944 10.1136/bmjgh-2017-000284 PMC5717944

[24] Steenstra I, de Bruin L, Mahood Q, et al. Prognostic factors for duration of sick leave in patients sick listed with acute low back pain: an update of a systematic review of the literature. Occup Environ Med. 2011;68:A74–5. 10.1136/oem.2004.015842 PMC1740930 16299094

[25] Üstün TB, Kostanjsek N, Chatterji S, et al. Measuring health and disability: Manual for WHO Disability Assessment Schedule WHODAS 2.0. World Health Organization; 2010. https://www.who.int/publications/i/item/measuring-health-and-disability-manual-for-who-disability-assessment-schedule-(-whodas-2.0)

[26] Üstün TB, Chatterji S, Kostanjsek N, et al. Developing the World Health Organization Disability Assessment Schedule 2.0. Bull World Health Organ. 2010;88:815–23. 21076562 10.2471/BLT.09.067231 PMC2971503

[27] Igwesi-Chidobe CN, Kitchen S, Sorinola IO et al. World Health Organisation Disability Assessment Schedule (WHODAS 2.0): development and validation of the Nigerian Igbo version in patients with chronic low back pain. BMC Musculoskelet Disord. 2020;21:755. 10.1186/s12891-020-03763-8 33203410 10.1186/s12891-020-03763-8 PMC7670680

[28] Smeets R, Köke A, Lin C, et al. Measures of function in low back pain/disorders: Low Back Pain rating SCale (LBPRS), Oswestry Disability Index (ODI), Progressive Isoinertial Lifting Evaluation (PILE), Quebec Back Pain Disability Scale (QBPDS), and Roland-Morris Disability Questionnaire. Arthritis Care Res (Hoboken). 2011;63:S158–S173. 22588742 10.1002/acr.20542

[29] Roland M, Morris R. A study of the natural history of back pain: part 1: development of a reliable and sensitive measure of disability in low-back pain. Spine (Phila Pa 1976). 10.1097/00007632-198303000-00004 6222486

[30] Roland M, Fairbank J. The Roland–Morris Disability Questionnaire and the Oswestry Disability Questionnaire. Spine. 2000;25:3115–24. 11124727 10.1097/00007632-200012150-00006

[31] Sigl T, Cieza A, Brockow T, et al. Content comparison of low back pain-specific measures based on the International Classification of Functioning, disability and health (ICF). Clin J Pain. 2006;22:147–53. 16428948 10.1097/01.ajp.0000155344.22064.f4

[32] Igwesi-Chidobe CN, Obiekwe C, Sorinola IO et al. Assessing self-reported disability in a low-literate population with chronic low back pain: cross-cultural adaptation and psychometric testing of Igbo Roland Morris Disability Questionnaire. *Disabil Rehabil*; 41. Epub ahead of print 2019. 10.1080/09638288.2017.1416185. 10.1080/09638288.2017.1416185 29239235

[33] Strand LI, Moe-Nilssen R, Ljunggren AE. Back Performance Scale for the assessment of mobility-related activities in people with back pain. Phys Ther. 2002;82:1213–23. 12444880

[34] Magnussen L, Strand LI, Lygren H. Reliability and validity of the Back Performance Scale: observing activity limitation in patients with back pain. Spine (Phila Pa 1976). 2004;29:903–7. 15082994 10.1097/00007632-200404150-00017

[35] Myklebust M, Magnussen L, Inger Strand L. Back Performance Scale scores in people without back pain: normative data. Adv Physiother. 2007;9:2–9.

[36] Hawker GA, Mian S, Kendzerska T, et al. Measures of adult pain: visual analog scale for pain (vas pain), numeric rating scale for pain (nrs pain), mcgill pain questionnaire (mpq), short-form mcgill pain questionnaire (sf‐mpq), chronic pain grade scale (cpgs), short form‐36 bodily pain scale. Arthritis Care Res (Hoboken). 2011;63(11):S240–S252. 22588748 10.1002/acr.20543

[37] Jensen MP, Karoly P, Braver S. The measurement of clinical pain intensity: a comparison of six methods. Pain. 1986;27:117–26. 3785962 10.1016/0304-3959(86)90228-9

[38] Jensen MP, McFarland CA. Increasing the reliability and validity of pain intensity measurement in chronic pain patients. Pain. 1993;55:195–203. 8309709 10.1016/0304-3959(93)90148-I

[39] Rodriguez CS. Pain measurement in the elderly: a review. Pain Manag Nurs. 2001;2:38–46. 11706769 10.1053/jpmn.2001.23746

[40] Igwesi-Chidobe CN. Development and preliminary evaluation of a self-management programme for people with non-specific chronic low back pain in rural Nigeria.

[41] Zimet GD, Dahlem NW, Zimet SG, et al. The Multidimensional Scale of Perceived Social Support. J Pers Assess. 1988;52:30–41.

[42] Zimet GD, Powell SS, Farley GK, et al. Psychometric characteristics of the Multidimensional Scale of Perceived Social Support. J Pers Assess. 1990;55:610–7. 2280326 10.1080/00223891.1990.9674095

[43] Igwesi-Chidobe CN, Kitchen S, Sorinola IO, et al. Adaptation and validation of the Nigerian Igbo Multidimensional Scale of Perceived Social Support in patients with chronic low back pain. Meas Instruments Soc Sci. 2021;3:1–15.

[44] Beaton DE, Bombardier C, Guillemin F, et al. Guidelines for the process of cross-cultural adaptation of self-report measures. Spine (Phila Pa 1976). 2000;25:3186–91. 11124735 10.1097/00007632-200012150-00014

[45] Wolcott MD, Lobczowski NG. Using cognitive interviews and think-aloud protocols to understand thought processes. Curr Pharm Teach Learn. 2021;13:181–8. 33454077 10.1016/j.cptl.2020.09.005

[46] Padilla J-L, Leighton JP. Cognitive interviewing and think aloud methods. Underst Investig Response Process Valid Res 2017; 211–28.

[47] Sim J, Wright CC. The kappa statistic in reliability studies: use, interpretation, and sample size requirements. Phys Ther. 2005;85:257–68. 15733050

[48] Webster K, Cella D, Yost K. The Functional Assessment of Chronic Illness Therapy (FACIT) measurement system: properties, applications, and interpretation. Health Qual Life Outcomes. 2003;1:1–7. 14678568 10.1186/1477-7525-1-79 PMC317391

[49] Hallal PC, Gomez LF, Parra DC, et al. Lessons learned after 10 years of IPAQ use in Brazil and Colombia. J Phys Act Heal. 2010;7:259–S264. 10.1123/jpah.7.s2.s259 20702914

[50] Weiss BD, Mays MZ, Martz W, et al. Quick assessment of literacy in primary care: the newest vital sign. Ann Fam Med. 2005;3:514–22. 16338915 10.1370/afm.405 PMC1466931

[51] Hahn EA, Cella D. Health outcomes assessment in vulnerable populations: measurement challenges and recommendations. Arch Phys Med Rehabil. 2003;84:35–S42. 10.1053/apmr.2003.50245 12692770

[52] Risser J, Jacobson TA, Kripalani S. Development and psychometric evaluation of the Self-Efficacy for Appropriate Medication Use Scale (SEAMS) in low-literacy patients with chronic disease. J Nurs Meas. 2007;15:203–19. 18232619 10.1891/106137407783095757

[53] McHugh ML. Interrater reliability: the Kappa statistic. Biochem Med. 2012;22:276–82. PMC3900052 23092060

[54] Shrout PE, Fleiss JL. Intraclass correlations: uses in assessing rater reliability. Psychol Bull. 1979;86:420. 18839484 10.1037//0033-2909.86.2.420

[55] Grotle M, Brox JI, Vollestad NK. Cross-cultural adaptation of the Norwegian versions of the Roland-Morris disability questionnaire and the Oswestry Disability Index. J Rehabil Med. 2003;35:241–7. 14582557 10.1080/16501970306094

[56] Tavakol M, Dennick R. Making sense of Cronbach’s alpha. Int J Med Educ. 2011;2:53. 28029643 10.5116/ijme.4dfb.8dfd PMC4205511

[57] Strauss ME, Smith GT. Construct validity: advances in theory and methodology. Annu Rev Clin Psychol. 2009;5:1–25. 19086835 10.1146/annurev.clinpsy.032408.153639 PMC2739261

[58] Igwesi-Chidobe CN, Sorinola IO, Godfrey EL. Only two subscales of the coping strategies Questionnaire are culturally relevant for people with chronic low back pain in Nigerian Igbo populations: a cross-cultural adaptation and validation study. J Patient-Reported Outcomes. 2021;5:1–16. 10.1186/s41687-021-00367-1 PMC8426442 34495431

[59] Morris LD, Grimmer-Somers KA, Louw QA, et al. Cross-cultural adaptation and validation of the South African Pain Catastrophizing Scale (SA-PCS) among patients with fibromyalgia. Health Qual Life Outcomes. 2012;10:137. 23173637 10.1186/1477-7525-10-137 PMC3548716

[60] Cohen J. Set correlation and contingency tables. Appl Psychol Meas. 1988;12:425–34.

[61] Tavakol M, Wetzel A. Factor analysis: a means for theory and instrument development in support of construct validity. Int J Med Educ. 2020;11:245. 33170146 10.5116/ijme.5f96.0f4a PMC7883798

[62] Nwachukwu PA. Towards an Igbo literary standard. Routledge; 2021.

[63] Omolayo AOOAO, Chinyere MHKAS. A comparative comparative comparative study of counting system in Hausa Study of Counting System in Hausa Study of Counting System in Hausa, Igbo and Igbo and Igbo and Yoruba. Int J Educ Res Manag Technol. 2019;4:30–4.

[64] Anyachebelu AL, Ezesinachi JN. Loans in Igbo Literature: a stylistics analysis of selected literary works. Niger J African Stud. 2021;3:214–27.

[65] van Vuuren B, Zinzen E, Van Heerden HJ, et al. Work and family support systems and the prevalence of lower back problems in a South African steel industry. J Occup Rehabil. 2007;17:409–21. 17636456 10.1007/s10926-007-9092-1

[66] Cheung K, Gillen M, Faucett J, et al. The prevalence of and risk factors for back pain among home care nursing personnel in Hong Kong. Am J Ind Med. 2006;49:14–22. 16362937 10.1002/ajim.20243

[67] Daniels C, Huang GD, Feuerstein M, et al. Self-report measure of low back-related biomechanical exposures: clinical validation. J Occup Rehabil. 2005;15:113–28. 15844672 10.1007/s10926-005-1214-z

[68] Ramezani M, Pourghayoomi E, Taghizadeh G. Job Requirements and Physical Demands (JRPD) questionnaire: cross-cultural adaptation and psychometric evaluation in Iranian Army personnel with chronic low back pain. BMC Musculoskelet Disord. 2022;23:1–9. 34986825 10.1186/s12891-021-04961-8 PMC8734355

[69] Pope DP, Silman AJ, Cherry NM, et al. Validity of a self-completed questionnaire measuring the physical demands of work. Scand J Work Environ Health. 1998;24(5):376–85. 9869309 10.5271/sjweh.358

[70] Angelo RL, Ryu RKN, Pedowitz RA, et al. Metric development for an arthroscopic Bankart procedure: assessment of face and content validity. Arthrosc J Arthrosc Relat Surg. 2015;31:1430–40. 10.1016/j.arthro.2015.04.093 26239785

[71] Knapp TR, Kimble LP, Dunbar SB. Distinguishing between the stability of a construct and the stability of an instrument in trait/state measurement. Nurs Res. 1998;47:60–2. 9478186 10.1097/00006199-199801000-00011

[72] Lodder P, Kupper N, Mols F, et al. Assessing the temporal stability of psychological constructs: an illustration of type D personality, anxiety and depression. J Res Pers. 2022;101:104299.

[73] Hartvigsen J, Hancock MJ, Kongsted A, et al. What low back pain is and why we need to pay attention. Lancet. 2018;391:2356–67. 29573870 10.1016/S0140-6736(18)30480-X

[74] Buchbinder R, van Tulder M, Öberg B, et al. Low back pain: a call for action. Lancet. 2018;391:2384–8. 29573871 10.1016/S0140-6736(18)30488-4

[75] Foster NE, Anema JR, Cherkin D, et al. Prevention and treatment of low back pain: evidence, challenges, and promising directions. Lancet. 2018;391:2368–83. 29573872 10.1016/S0140-6736(18)30489-6

