# Supplementary material for: Challenges of Measuring Self-Reported Exposure to Occupational Biomechanical Risk Factors Amongst People with Low Literacy Engaged in Manual Labour: Findings from a Cross-Cultural Adaptation and Psychometric Investigation in an African Population with Chronic Low Back Pain
Source: J Occup Rehabil. 2024 Feb 20;34(4):847–62. doi: 10.1007/s10926-024-10171-5 (PMC11550223; doi:10.1007/s10926-024-10171-5)
Supplement: Supplementary file 1 — Supplementary material 1 (DOCX 23.9 kb) [file 10926_2024_10171_MOESM1_ESM.docx]

**IGBO OCCUPATIONAL RISK FACTOR QUESTIONNAIRE (IGBO-ORFQ)**

Ka anyi wee nwee ike i nyere gi aka nke oma, o di mkpa ka anyi mara maka oru I na aru. Biko zaa ajuju ndi aga aju gi. Biko zaa ha nile.

**Biko kowaa ihe bu isi ihe di na oru I na aru**

............................................................................................................................................

**Biko zaa EE ma o bu MBA iji zaa ajuju ndi a n’otu na otu**

1. I nwere ike I nara ezumike maka ubochi o bula na oru gi tinyere ezumike nke eweputara gi n’ime ubochi o bula?

Ee Mba

1. I na achoputakari na o nwere mgbe I nweghi ike iru oru n’ihi ihe ndaputa, dika mgbe igwe eji aru oru mebiri ma o bu na ngwa oru adighi?

Ee Mba

1. I nwere ike ichikwa na ihazicha etu oru si agara gi?

Ee Mba

1. Nchikwa na nhazi oru gi o dabere na ihe ndi ozo (dika igwe oru machine, igwe computer, ndi azuma ahia)

Ee Mba

1. I na aru oru na onodu nmanye dika oge, ezughi ike ma o bu ngwa ngwa I gbanari oge?

Ee Mba

**Oge ole ka o na ewe gi ime ihe ndi a n’ulo oru, inodu ala/ma o bu ikwuru oto**

1. Ihuru ala ntakiri, aka gi agaghi eru ikpere gi
   - 1. **Oraka ahu/o foro nta ka o buru ihe anaghi eme eme**

- - 1. Ihe dika pacenti iri na oge oru

(ma o bu ihe kariri okara otu ubochi n’ime otu izu uka nwere ubochi asaa)

- - 1. Ihe dika pacenti iri abuo na ise na oge oru

(ma o bu otu ubochi na ihe kariri okara n’ime otu izu uka nwere ubochi asaa)

- - 1. **Ihe dika okara oge oru n’ime otu izu uka nwere ubochi asaa**

- - 1. Ihe dika pacenti iri asaa na ise na oge oru

(ma o bu ubochi ise na ihe erughi okara otu ubochi na ime otu izu uka nwere ubochi asaa)

- - 1. **O foro ntakiri ka o buru mgbe nile**

1. Ihuru ala ka aka gi gafee ikpere gi
   - 1. **Oraka ahu/o foro nta ka o buru ihe anaghi eme eme**

- - 1. Ihe dika pacenti iri na oge oru

(ma o bu ihe kariri okara otu ubochi n’ime otu izu uka nwere ubochi asaa)

- - 1. Ihe dika pacenti iri abuo na ise na oge oru

(ma o bu otu ubochi na ihe kariri okara n’ime otu izu uka nwere ubochi asaa)

- - 1. **Ihe dika okara oge oru n’ime otu izu uka nwere ubochi asaa**

- - 1. Ihe dika pacenti iri asaa na ise na oge oru

(ma o bu ubochi ise na ihe erughi okara otu ubochi na ime otu izu uka nwere ubochi asaa)

- - 1. **O foro ntakiri ka o buru mgbe nile**

1. Inyaghari ma o bu ihighari etiti ahu karia ihe dika digrii iri ano na ise na ihulata gaa na akuku
   - 1. **Oraka ahu/o foro nta ka o buru ihe anaghi eme eme**

- - 1. Ihe dika pacenti iri na oge oru

(ma o bu ihe kariri okara otu ubochi n’ime otu izu uka nwere ubochi asaa)

- - 1. Ihe dika pacenti iri abuo na ise na oge oru

(ma o bu otu ubochi na ihe kariri okara n’ime otu izu uka nwere ubochi asaa)

- - 1. **Ihe dika okara oge oru n’ime otu izu uka nwere ubochi asaa**

- - 1. Ihe dika pacenti iri asaa na ise na oge oru

(ma o bu ubochi ise na ihe erughi okara otu ubochi na ime otu izu uka nwere ubochi asaa)

- - 1. **O foro ntakiri ka o buru mgbe nile**

**Ihe dika oge ole ka i na eji eme ihe ndi a n’ulo oru gi (kwuo iji gosi nke bu aziza gi ka e were akara gosi nke bu aziza gi n’oge edeputara maka nke o bula)**

1b. Ibulite ihe buru ibu na anyi aru ihe dika mgbati aka gi

- - 1. **Oraka ahu/o foro nta ka o buru ihe anaghi eme eme**

- - 1. Ihe dika pacenti iri na oge oru

(ma o bu ihe kariri okara otu ubochi n’ime otu izu uka nwere ubochi asaa)

- - 1. Ihe dika pacenti iri abuo na ise na oge oru

(ma o bu otu ubochi na ihe kariri okara n’ime otu izu uka nwere ubochi asaa)

- - 1. **Ihe dika okara oge oru n’ime otu izu uka nwere ubochi asaa**

- - 1. Ihe dika pacenti iri asaa na ise na oge oru

(ma o bu ubochi ise na ihe erughi okara otu ubochi na ime otu izu uka nwere ubochi asaa)

- - 1. **O foro ntakiri ka o buru mgbe nile**

2b. Iji otu aka buru ibu

- - 1. **Oraka ahu/o foro nta ka o buru ihe anaghi eme eme**

- - 1. Ihe dika pacenti iri na oge oru

(ma o bu ihe kariri okara otu ubochi n’ime otu izu uka nwere ubochi asaa)

- - 1. Ihe dika pacenti iri abuo na ise na oge oru

(ma o bu otu ubochi na ihe kariri okara n’ime otu izu uka nwere ubochi asaa)

- - 1. **Ihe dika okara oge oru n’ime otu izu uka nwere ubochi asaa**

- - 1. Ihe dika pacenti iri asaa na ise na oge oru

(ma o bu ubochi ise na ihe erughi okara otu ubochi na ime otu izu uka nwere ubochi asaa)

- - 1. **O foro ntakiri ka o buru mgbe nile**

3b. Ibughari ihe ndi na adighi mfe I jide aka ma na enweghikwa ebe a na-ejide ya aka

- - 1. **Oraka ahu/o foro nta ka o buru ihe anaghi eme eme**

- - 1. Ihe dika pacenti iri na oge oru

(ma o bu ihe kariri okara otu ubochi n’ime otu izu uka nwere ubochi asaa)

- - 1. Ihe dika pacenti iri abuo na ise na oge oru

(ma o bu otu ubochi na ihe kariri okara n’ime otu izu uka nwere ubochi asaa)

- - 1. **Ihe dika okara oge oru n’ime otu izu uka nwere ubochi asaa**

- - 1. Ihe dika pacenti iri asaa na ise na oge oru

(ma o bu ubochi ise na ihe erughi okara otu ubochi na ime otu izu uka nwere ubochi asaa)

- - 1. **O foro ntakiri ka o buru mgbe nile**

4b. I kwa ma o bu I doro ibu

- - 1. **Oraka ahu/o foro nta ka o buru ihe anaghi eme eme**

- - 1. Ihe dika pacenti iri na oge oru

(ma o bu ihe kariri okara otu ubochi n’ime otu izu uka nwere ubochi asaa)

- - 1. Ihe dika pacenti iri abuo na ise na oge oru

(ma o bu otu ubochi na ihe kariri okara n’ime otu izu uka nwere ubochi asaa)

- - 1. **Ihe dika okara oge oru n’ime otu izu uka nwere ubochi asaa**

- - 1. Ihe dika pacenti iri asaa na ise na oge oru

(ma o bu ubochi ise na ihe erughi okara otu ubochi na ime otu izu uka nwere ubochi asaa)

- - 1. **O foro ntakiri ka o buru mgbe nile**

5b. Ibu ihe di aro dika pound iri ruo iri ato ma o bu kilogram ise ruo iri na ano ma o bu ihe na anyi ka mmiri lita ise ruo lita iri na ano

- - 1. **Oraka ahu/o foro nta ka o buru ihe anaghi eme eme**

- - 1. Ihe dika pacenti iri na oge oru

(ma o bu ihe kariri okara otu ubochi n’ime otu izu uka nwere ubochi asaa)

- - 1. Ihe dika pacenti iri abuo na ise na oge oru

(ma o bu otu ubochi na ihe kariri okara n’ime otu izu uka nwere ubochi asaa)

- - 1. **Ihe dika okara oge oru n’ime otu izu uka nwere ubochi asaa**

- - 1. Ihe dika pacenti iri asaa na ise na oge oru

(ma o bu ubochi ise na ihe erughi okara otu ubochi na ime otu izu uka nwere ubochi asaa)

- - 1. **O foro ntakiri ka o buru mgbe nile**

6b. Ibu ihe di aro karia pound iri ato ma o bu kilogram iri na ano ma o bu ihe na anyi ka mmiri lita iri na ano

- - 1. **Oraka ahu/o foro nta ka o buru ihe anaghi eme eme**

- - 1. Ihe dika pacenti iri na oge oru

(ma o bu ihe kariri okara otu ubochi n’ime otu izu uka nwere ubochi asaa)

- - 1. Ihe dika pacenti iri abuo na ise na oge oru

(ma o bu otu ubochi na ihe kariri okara n’ime otu izu uka nwere ubochi asaa)

- - 1. **Ihe dika okara oge oru n’ime otu izu uka nwere ubochi asaa**

- - 1. Ihe dika pacenti iri asaa na ise na oge oru

(ma o bu ubochi ise na ihe erughi okara otu ubochi na ime otu izu uka nwere ubochi asaa)

- - 1. **O foro ntakiri ka o buru mgbe nile**

7b. I bu ibu kariri pound iri ma o bu kilogram ise/ihe na anyi ka mmiri lita ise gaa ruo ihe kariri feet iri ano ma o bu site na ulo gi ruo ulo agbata obi gi

- - 1. **Oraka ahu/o foro nta ka o buru ihe anaghi eme eme**

- - 1. Ihe dika pacenti iri na oge oru

(ma o bu ihe kariri okara otu ubochi n’ime otu izu uka nwere ubochi asaa)

- - 1. Ihe dika pacenti iri abuo na ise na oge oru

(ma o bu otu ubochi na ihe kariri okara n’ime otu izu uka nwere ubochi asaa)

- - 1. **Ihe dika okara oge oru n’ime otu izu uka nwere ubochi asaa**

- - 1. Ihe dika pacenti iri asaa na ise na oge oru

(ma o bu ubochi ise na ihe erughi okara otu ubochi na ime otu izu uka nwere ubochi asaa)

- - 1. **O foro ntakiri ka o buru mgbe nile**

8b. Inodu ala

- - 1. **Oraka ahu/o foro nta ka o buru ihe anaghi eme eme**

- - 1. Ihe dika pacenti iri na oge oru

(ma o bu ihe kariri okara otu ubochi n’ime otu izu uka nwere ubochi asaa)

- - 1. Ihe dika pacenti iri abuo na ise na oge oru

(ma o bu otu ubochi na ihe kariri okara n’ime otu izu uka nwere ubochi asaa)

- - 1. **Ihe dika okara oge oru n’ime otu izu uka nwere ubochi asaa**

- - 1. Ihe dika pacenti iri asaa na ise na oge oru

(ma o bu ubochi ise na ihe erughi okara otu ubochi na ime otu izu uka nwere ubochi asaa)

- - 1. **O foro ntakiri ka o buru mgbe nile**

1. I gbusa ikpere na ala, ma o bu ihuru ala
   - 1. **Oraka ahu/o foro nta ka o buru ihe anaghi eme eme**

- - 1. Ihe dika pacenti iri na oge oru

(ma o bu ihe kariri okara otu ubochi n’ime otu izu uka nwere ubochi asaa)

- - 1. Ihe dika pacenti iri abuo na ise na oge oru

(ma o bu otu ubochi na ihe kariri okara n’ime otu izu uka nwere ubochi asaa)

- - 1. **Ihe dika okara oge oru n’ime otu izu uka nwere ubochi asaa**

- - 1. Ihe dika pacenti iri asaa na ise na oge oru

(ma o bu ubochi ise na ihe erughi okara otu ubochi na ime otu izu uka nwere ubochi asaa)

- - 1. **O foro ntakiri ka o buru mgbe nile**

1. I rigoro ulo elu ma o bu elu obe/lada
   - 1. **Oraka ahu/o foro nta ka o buru ihe anaghi eme eme**

- - 1. Ihe dika pacenti iri na oge oru

(ma o bu ihe kariri okara otu ubochi n’ime otu izu uka nwere ubochi asaa)

- - 1. Ihe dika pacenti iri abuo na ise na oge oru

(ma o bu otu ubochi na ihe kariri okara n’ime otu izu uka nwere ubochi asaa)

- - 1. **Ihe dika okara oge oru n’ime otu izu uka nwere ubochi asaa**

- - 1. Ihe dika pacenti iri asaa na ise na oge oru

(ma o bu ubochi ise na ihe erughi okara otu ubochi na ime otu izu uka nwere ubochi asaa)

- - 1. **O foro ntakiri ka o buru mgbe nile**

1. Iji ngwa oru aka oku latric a na-ejide n’aka aru oru (dika drills, saws, jack hammer)
   - 1. **Oraka ahu/o foro nta ka o buru ihe anaghi eme eme**

- - 1. Ihe dika pacenti iri na oge oru

(ma o bu ihe kariri okara otu ubochi n’ime otu izu uka nwere ubochi asaa)

- - 1. Ihe dika pacenti iri abuo na ise na oge oru

(ma o bu otu ubochi na ihe kariri okara n’ime otu izu uka nwere ubochi asaa)

- - 1. **Ihe dika okara oge oru n’ime otu izu uka nwere ubochi asaa**

- - 1. Ihe dika pacenti iri asaa na ise na oge oru

(ma o bu ubochi ise na ihe erughi okara otu ubochi na ime otu izu uka nwere ubochi asaa)

- - 1. **O foro ntakiri ka o buru mgbe nile**

1. Inya ugbo ala ma o bu igwe ugbo ndi eji aru oru (gwongworo, ugbo ala ukwu, ugbo okporo igwe/ugbo oloko, forklifts)
   - 1. **Oraka ahu/o foro nta ka o buru ihe anaghi eme eme**

- - 1. Ihe dika pacenti iri na oge oru

(ma o bu ihe kariri okara otu ubochi n’ime otu izu uka nwere ubochi asaa)

- - 1. Ihe dika pacenti iri abuo na ise na oge oru

(ma o bu otu ubochi na ihe kariri okara n’ime otu izu uka nwere ubochi asaa)

- - 1. **Ihe dika okara oge oru n’ime otu izu uka nwere ubochi asaa**

- - 1. Ihe dika pacenti iri asaa na ise na oge oru

(ma o bu ubochi ise na ihe erughi okara otu ubochi na ime otu izu uka nwere ubochi asaa)

- - 1. **O foro ntakiri ka o buru mgbe nile**

1. Iru oru na ala na ami ami ma o bu ebe ala adighi larii
   - 1. **Oraka ahu/o foro nta ka o buru ihe anaghi eme eme**

- - 1. Ihe dika pacenti iri na oge oru

(ma o bu ihe kariri okara otu ubochi n’ime otu izu uka nwere ubochi asaa)

- - 1. Ihe dika pacenti iri abuo na ise na oge oru

(ma o bu otu ubochi na ihe kariri okara n’ime otu izu uka nwere ubochi asaa)

- - 1. **Ihe dika okara oge oru n’ime otu izu uka nwere ubochi asaa**

- - 1. Ihe dika pacenti iri asaa na ise na oge oru

(ma o bu ubochi ise na ihe erughi okara otu ubochi na ime otu izu uka nwere ubochi asaa)

- - 1. **O foro ntakiri ka o buru mgbe nile**

1. Iru oru n’elu ihe/ebe di elu (dika n’elu ulo, lada, obe na ndi ozo)
   - 1. **Oraka ahu/o foro nta ka o buru ihe anaghi eme eme**

- - 1. Ihe dika pacenti iri na oge oru

(ma o bu ihe kariri okara otu ubochi n’ime otu izu uka nwere ubochi asaa)

- - 1. Ihe dika pacenti iri abuo na ise na oge oru

(ma o bu otu ubochi na ihe kariri okara n’ime otu izu uka nwere ubochi asaa)

- - 1. **Ihe dika okara oge oru n’ime otu izu uka nwere ubochi asaa**

- - 1. Ihe dika pacenti iri asaa na ise na oge oru

(ma o bu ubochi ise na ihe erughi okara otu ubochi na ime otu izu uka nwere ubochi asaa)

- - 1. **O foro ntakiri ka o buru mgbe nile**

**Ugboro ole ka i na ebuli ihe di aro dika nke:**

1. Ihe na erughi pound iri ma o bu kilogram ise ma o bu ihe na anyi ka mmiri lita ise
2. Oraka ahu/o foro nta ka o buru ihe anaghi eme eme
3. orughi otu ugboro n’ime otu awa
4. site otu ugboro rue ugboro iri n’ime otu awa
5. site iri na otu ruo ugboro iri ato n’ime otu awa
6. ihe kariri ugboro iri ato n’ime otu awa.
7. Ihe di pound iri ruo pound iri ato ma o bu kilogram ise ruo kilogram iri na ano ma o bu ihe na anyi ka mmiri lita ise ruo mmiri lita iri na ano
   - 1. Oraka ahu/o foro nta ka o buru ihe anaghi eme eme
     2. orughi otu ugboro n’ime otu awa
     3. site otu ugboro rue ugboro iri n’ime otu awa
     4. site iri na otu ruo ugboro iri ato n’ime otu awa
     5. ihe kariri ugboro iri ato n’ime otu awa.
8. Ihe kariri pound iri ato ma o bu kilogram iri na ano ma o bu ihe na anyi ka mmiri lita iri na ano
   - 1. Oraka ahu/o foro nta ka o buru ihe anaghi eme eme
     2. orughi otu ugboro n’ime otu awa
     3. site otu ugboro rue ugboro iri n’ime otu awa
     4. site iri na otu ruo ugboro iri ato n’ime otu awa
     5. ihe kariri ugboro iri ato n’ime otu awa.

**Imeela maka oge i nyere anyi!**
